# Eating disorders in medical students: prevalence, risk factors, comparison with the general population

**DOI:** 10.3389/fpsyg.2024.1515084

**Published:** 2025-01-07

**Authors:** Raluca Motorga, Mihaela Ionescu, Florina Nechita, Daniela Micu, Iulia Băluțoiu, Maria Mădălina Dinu, Dan Nechita

**Affiliations:** ^1^Department of Behavioral Sciences, University of Medicine and Pharmacy of Craiova, Craiova, Romania; ^2^Department of Medical Informatics and Biostatistics, University of Medicine and Pharmacy of Craiova, Craiova, Romania; ^3^Department of Foreign Languages, University of Medicine and Pharmacy of Craiova, Craiova, Romania

**Keywords:** eating disorders, medical students, social/family pressure, BMI, sleep, thin body, anorexia, bulimia

## Abstract

**Objectives:**

The main objectives were to investigate the prevalence of ED and associated risk factors among medical students in Romania, as well as to determine which variables may predict ED and to explore the differences between medical students and the general population.

**Methods:**

The Eating Disorders Inventory questionnaire (EDI-3) was applied. Also, the body mass index of the students was calculated, socio-demographic information regarding personal and family medical history was collected (mental and chronic diseases, self-reported sleep difficulties in the past 6 months, family history of obesity) and potentially risky events (history of ridicule, major negative events, social pressure to be thin from family, friends, media).

**Results:**

37.1% students are at risk of eating disorders, 41% females and 23% males, according to the EDI-3. 14.42% were underweight, 65.05% normal weight, 16.99% overweight and 3.53% obese. Compared to the nationally representative sample in Romania, medical students had significantly higher scores for ED risk and for all other psychological factors.

**Conclusion:**

Medical students have a high risk of developing ED, even higher than the general population. Several factors were associated with the ED risk, including female gender, experiencing sleeping difficulties, feelings of ridicule, family pressure and media pressure, prior ED history, high BMI, negative events and family history of mental illness. The regression analysis showed that family pressure is a strong predictor for ED risk.

## 1 Introduction

Eating disorders (ED) are complex mental health conditions characterized by unhealthy behavior and attitude toward food, eating and body weight, which, if not effectively treated, may have serious, even fatal, physical and psycho-emotional consequences (Arcelus et al., 2011). Each type of eating disorder has its own symptoms and diagnostic criteria, sometimes even similar or overlapping. These disorders can affect individuals of all ages, gender, ethnicities, backgrounds and they generally begin in adolescence. Therefore, understanding the nature and the influence of eating disorders is crucial to identifying and effectively addressing these problems. The most common types of eating disorders include anorexia nervosa (with its two forms: restrictive—AN-R and compulsive/purge eating—AN-AC/P), bulimia nervosa (BN), compulsive/binge eating disorder (BED 5) (American Psychiatric Association, 2016).

The Biopsychosocial model is a theoretical framework that offers a complex understanding of the ED by including biological, psychological and social factors and their specific interaction. This approach suggests the complexity of these mental conditions and emphasizes that they cannot be explained by only a single factor. From the point of view of the biological factors, research suggests that genetics (Watson et al., 2023) and being of female gender (Culbert et al., 2021) can increase the risk of developing ED. Family history of ED also has an important role (Chapman et al., 2021). The psychological factors suggests that there are some personality characteristics, like perfectionism (Stackpole et al., 2023), neuroticism and low self-esteem (Cervera et al., 2003), impulsivity and compulsivity (Solly et al., 2023) that can lead to dysfunctional coping strategies, thus increasing and/or maintaining the risk of ED. Also, ED can manifest with other mental health conditions, like anxiety, depression hazardous/harmful alcohol use and others (Solly et al., 2023). In the literature, we can find evidence of how important is the social factor in shaping attitudes toward eating disorders and/or body image (Cepeda-Benito and Moreno-Domínguez, 2019). Gender, socioeconomic status, ethnicity, body image and social pressure and performance sport are some of the social factors that can influence the development and persistence of the ED (Barakat et al., 2023). Despite the belief of many in the past decades that eating disorders were specific to Western culture (Baker et al., 2009; Bould et al., 2015), research has shown that in non-Western countries these conditions are not exclusive to Western culture (Azzouzi et al., 2019). Also, studies showed that eating disorders prevalence in non-Western countries is lower than in the Western countries, but it tends to grow (Cena et al., 2022; Soh and Walter, 2013). Furthermore, Western media and the exposure to an ideal body shape, as well the impact of globalization, have played significant roles in the increasing of eating disorders at global level (Pike et al., 2014; Jahrami et al., 2019). In conclusion, a better understanding of the interaction of these factors can lead to more effective programs of prevention and treatment, by creating more personalized and cultural relevant programs for those who need it.

Of significant concern is the prevalence of eating disorders among medical students population worldwide, as shown by many research studies. It is well-known that medical universities are rigorous and that medical students present a significant risk in developing a series of psychological issues as high anxiety (Nechita et al., 2018; Nechita et al., 2019), high levels of stress, depressive symptoms (Petreanu et al., 2017), but also eating disorders, as a result of the pressures during the faculty, such as the large volume of information to be memorized in a relatively short time, the combination of theoretical and practical aspects, etc. (Jahrami et al., 2019). A systematic review and a meta-analysis showed that the prevalence of eating disorders risk among medical students was estimated by 10.4% globally, based on data from 19 cross-sectional studies in nine countries, which involved a total of 5,722 participants. Prevalence varied between studies, from 2.2% to 29.1% (Bizri et al., 2020; Fekih-Romdhane et al., 2022). Additionally, a cross-sectional survey conducted at King Abdul-Aziz University in Saudi Arabia found that among 417 medical students, the prevalence of eating disorders was 32.1%. The study found that preclinical female students and the ones that were overweight or obese were significantly more likely to be associated with a high risk of eating disorders. This emphasizes the importance of addressing this health issue among medical students and of implementing preventive programs, particularly targeting female medical school students (Ghamri et al., 2022). Eating disorders prevalence among medical students is a concern that justifies future research and the implementation of prevention and treatment strategies adapted to this population. These results highlight the need for proactive measures to support the mental and emotional wellbeing of medical students and reduce the risk of developing eating disorders during their training (Jahrami et al., 2019).

Studies conducted in Romania, although few in number, showed that eating disorders are widespread, with observed differences between ethnicities and a significant influence of Western values on the prevalence of these disorders (Kovács Krizbai and Szabó, 2009; Kovács, 2007; Joja et al., 2015). Prevalence for Romanian Saxons, students in general school is AN = 0.3% and BN = 0.8% in females, with cultural differences (0.5% Saxons and 0.3% for Romanian girls) (Kovács Krizbai and Szabó, 2009). It has been observed that young females tend to have a lower BMI and higher concerns about weight, without always showing body dissatisfaction (Joja et al., 2015).

A team of experts in mental health from Romania conducted a study in 2023 that aimed to describe the situation of the mental health as it is in Romania nowadays. Some of their most worrying conclusions included: insufficient and dated information of the prevalence of mental disorders in Romania (more than 17 years ago), lack of prioritizing of mental health in the state politics, underfunding of this medical branch, fewer number of specialists than in the European Union countries and that stigma and poverty makes hard for the people to access specialized treatments. The authors argued that this may lead to discrepancies compared to the other countries (Bartuc et al., 2023). The authors of the same study revealed that there are differences in ED prevalence in Romania and USA and other developed countries, possibly due to underdiagnosing/over-reporting of symptoms (Bartuc et al., 2023).

Research in Romania concerning eating disorder risk among medical students is limited, highlighting the need for further studies in this demographic, especially in the context of stigma and the few resources available that complicates the situation (Bartuc et al., 2023). The few existing studies indicate varying prevalence rates of ED, alongside identified risk factors and associations with academic and lifestyle behaviors that included: being overweight associated with poor academic performance (Petreanu et al., 2017), smoking, and excessive alcohol consumption; the stress associated with the intensive study and the high academic demands of the medical field; increased concern for physical appearance and the aesthetic implications of the dental profession; some personality traits such as perfectionism and fear of failure, frequently found among students in dental medicine; the significant association between ED and dietary practices among medical and pharmacy students (Brumboiu et al., 2018; Iorga et al., 2018; Mocanu, 2013).

Considering the high demands the medical students have to cope with during their education, and the fact that eating disorders have severe consequences, an analysis of the prevalence of ED among medical students’ population is very important, especially considering the context of Romania’s mental health system. Also, the prevalence of ED among medical students in Romania might be higher in comparison with the general population (Petreanu et al., 2017).

Therefore, it is absolutely necessary to do a screening for ED risk among medical students population and also to identify the ED risk factors and to compare the prevalence with the general population, in order to have an in-depth analysis of the situation as presented at the University of Medicine and Pharmacy of Craiova. By addressing this issue, we aim also to help medical universities to develop and implement strategies that better respond to their medical students’ needs.

## 2 Materials and methods

### 2.1 Participants and procedure

This is a cross-sectional study that involved 312 students from a prestigious university of medicine in Romania. The participation was voluntary. The minimum sample size was computed using G*Power 3.1.9.7, Heinrich Heine University Düsseldorf, Germany, considering a significance level *α* of 0.05, a power 1 − *β* equal to 0.90, and a medium effect size value of 0.5 (keeping in mind an awareness of practical significance), resulting in a study lot of at least 278 participants.

Conducted between October 2023 and January 2024, this study was a broad analysis of the characteristics and the relationships between different aspects. The research participants were from years I to V, thus representing a relevant variety of experience and academic development. There were no participants from the last year of study (year VI).

The questionnaire was applied online through Google Forms Platform and was distributed either in class or accessible directly from the university website. This contained Eating Disorder Inventory-3 (EDI-3) and a socio-demographic questionnaire. To complete the questionnaire, all questions had to be answered. In the part of the questionnaire completion instructions, the participants were informed that the completed data are anonymous, confidential, and that the completion of the questionnaire represents the GDPR consent to the collection of personal data, such as age, gender, physical parameters (weight, height), etc. Participation was voluntary and no rewards were offered for involvement in the study. They were also told that the answers and the participation in the study would have no influence on their grades.

The study was conducted in accordance with the Declaration of Helsinki and approved by the Ethics Committee of the University of Medicine and Pharmacy of Craiova, no. 174/14.09.2023. The participants provided their written informed consent to participate in this study.

### 2.2 Data collection

For our research, we opted for the EDI-3 (Eating Disorder Inventory-3), in the long form, with 91 items. It is a self-report questionnaire developed by Garner (2004), which serves as a standardized clinical assessment of the symptoms associated with eating disorders (Garner, 2010). It is designed to assess the presence of eating disorders, including anorexia nervosa, bulimia nervosa and binge eating disorder, as well as associated psycho-logical traits or constructs. The questionnaire contains 91 items organized into 12 primary scales, consisting of three scales specific to eating disorders and nine scales referring to general psychological constructs, important in the emergence, manifestation and persistence of the ED and generate six composite scales. The EDI-3 questionnaire shows excellent reliability and validity. It was adapted and standardized on the Romanian population by Miclea et al. (2011).

Also, the body mass index (BMI) of the students was computed, socio-demographic information was collected, as well as data related to personal and family medical history (mental disorders, chronic diseases, presence of sleep difficulties in the past 6 months, family history of obesity) and potentially risk events (feelings of ridicule, major negative events, social pressure to be thin from family, friends, media).

Clinical data included the calculation of BMI: weight/height^2^. The weight was expressed in kilograms and the height in centimeters. The participants were than divided into underweight (<18.5), normal weight (between 18.5 and <25), overweight (25 and <30) and obese (30 and <35 = group I; 35 and <40 = group II; > 40 = morbid obesity), according to World Health Organization.

### 2.3 Statistical analysis

All responses collected from the questionnaires were initially centralized using Microsoft Excel (San Francisco, USA). Continuous variables were defined as mean ± standard deviation (mean ± SD) and they were compared using the following tests: Kendall’s tau-b test or the Mann–Whitney U test for non-Gaussian distributions. Nominal variables were expressed as numerical values and corresponding percentages, and their association was assessed with either the Chi-square test or the Fisher Exact test. All statistical tests were applied using the Statistical Package for Social Sciences (SPSS) program, version 26 (IBM Corp., USA). Part of the students’ answers were included as input data in a binomial logistic regression model, which evaluated the probability of occurrence corresponding to the risk of developing ED in relation to the following variables: gender, age, residence, marital status, advanced studies (college), self-reported sleep difficulties, pressure to be thin expressed by family members but also by society, personal familial history of chronic diseases, mental illness and weight control difficulties, presence of major negative events, BMI, reporting the feeling of being ridiculed. The *α*-threshold was set at 5% and *p*-value less than 0.05 was considered statistically significant.

## 3 Results

### 3.1 Study group description

The study was conducted on a sample of 312 students from a prestigious university of medicine in Romania. Among them, 21.79% were males and 78.21% were females, with a mean age of 20.28 ± 3.714 years (range 18–46 years). There are less men than women included in the study, because women represent the majority of students enrolled at the University of Medicine and Pharmacy of Craiova (UMFCv)—over 70%. The participants attended three different faculties within UMFCv, Romania, namely General Medicine, Dental Medicine, and Midwives and Nurses, being enrolled in the 1st to 5th year of study. The majority (73.72%) had urban residence, and 77.56% had an average income level. Regarding marital status, approximately 42% were in a relationship, while 58% were single (Tables 1–4).

**Table 1:** Distribution of participants according to socio-demographic parameters.

Parameter	Category	*n*	*n*%
Gender	Male	68	21.79%
	Female	244	78.21%
Age	<19 years old	184	58.97%
	≥20 years old	128	41.03%
	Minimum	18	–
	Maximum	46	–
	Mean ± SD	20.28 ± 3.714	–
Faculty	General Medicine	180	57.69%
	Dental Medicine	49	15.71%
	Midwives and Nurses	83	26.60%
Year of study	1	240	76.92%
	2	37	11.86%
	3	20	6.41%
	4	13	4.17%
	5	2	0.64%
Marital status	Single	181	58.01%
	In a relationship	131	41.99%
Residence	Urban	230	73.72%
	Rural	82	26.28%
Perceived income level	Low	42	13.46%
	Average	242	77.56%
	High	28	8.97%

**Table 2:** Distribution of participants by gender and BMI category.

Category	Males	Females	Total
Underweight	1 (2.2%)	44 (97.8%)	45 (100%)
Normal weight	45 (22.2%)	158 (77.8%)	203 (100%)
Overweight	19 (35.8%)	34 (64.2%)	53 (100%)
Obese	3 (27.3%)	8 (72.7%)	11 (100%)

**Table 3:** Distribution of participants by health and wellbeing.

Parameter	Category	*n*	*n*%
Self-reported sleep difficulties in the last 6 months^1^	Yes	186	59.62%
	No	126	40.38%
Weight/appearance ridicule	Never	84	26.92%
	Rarely	84	26.92%
	Sometimes	62	19.87%
	Often	32	10.26%
	Usually	16	5.13%
	Always	34	10.90%
Family history of difficulty maintaining a normal weight/ED	Yes	158	50.64%
	No	154	49.36%
Personal history of chronic disease	Yes	20	6.41%
	No	292	93.59%
Family history of chronic disease	Yes	124	39.74%
	No	188	60.26%
Family history of mental disease	Yes	43	13.78%
	No	269	86.22%
Major events with negative impact	Never	32	10.26%
	Rarely	109	34.94%
	Sometimes	116	37.18%
	Often	38	12.18%
	Usually	14	4.49%
	Always	3	0.96%
BMI	Underweight	45	14.42%
	Normal weight	203	65.05%
	Overweight	53	16.99%
	Obese	11	3.53%
	Minimum	15.62	–
	Maximum	39.79	–
	Mean	22.251	–
	SD^*^	3.857	–
Desired weight	Satisfied with actual weight	146	46.79%
	5 kg more	27	8.65%
	5 kg less	58	18.59%
	Over 10 kg less	72	23.08%
	Over 10 kg more	9	2.88%

1 Self-reported of sleep difficulties in the last 6 months.

**Table 4:** Distribution of participants by perception of the social pressure.

Parameter	Category	*n*	*n*%
Pressure to be thin from friends/family	Never	107	34.29%
	Rarely	63	20.19%
	Sometimes	64	20.51%
	Often	30	9.62%
	Usually	18	5.77%
	Always	30	9.62%
Pressure to be thin from media	Never	101	32.37%
	Rarely	60	19.23%
	Sometimes	57	18.27%
	Often	34	10.90%
	Usually	21	6.73%
	Always	39	12.50%

### 3.2 Health and wellbeing outcomes

The BMI analysis revealed that 14.42% of the respondents were underweight (F = 44/97.8% vs. M = 2.2%), 65.05% had normal weight (F = 158/77.8% vs. M = 45/22.2%), 16.99% were overweight (F = 34/64.2% vs. M = 19/35.8%) and 3.53% were obese (F = 8/72.7% vs. M = 3/27.3%). The distribution according to the respondents’ gender is emphasized in Table 2.

More than half of the participants (59.62%) self-reported the presence of sleep difficulties. There are differences between women and men when it comes to sleep difficulties. The association between gender and the existence of sleep difficulties reported in the questionnaires was analyzed based on a Chi-square test. All frequencies were greater than 5. A statistically significant association was identified between gender and the existence of sleep difficulties, *χ*^2^(1) = 4.439, *p* = 0.035. More than half of females (62.7%) reported having sleep difficulties in the past 6 months, compared to only 48.5% of males who reported similar problems. A moderate association was identified between these parameters, *φ* = 0.322, *p* = 0.035. Also, 53.84% of students reported that they were rarely or never ridiculed, while 26.29% experienced the feeling of being ridiculed often, usually or even always. Within the group of students who felt ridiculed often, usually or always, there were 26.2% females, compared to only 19.1% males. Thus, there is a statistically significant moderate association between gender and the feeling of being ridiculed, *χ*^2^(1) = 14.455, *φ* = 0.215, *p* = 0.013.

Most students who always felt pressure to be thin from family members (63.3%), also felt that they were always ridiculed, or often ridiculed (16.7%). Similar results were obtained for the other responses—the more pressure from family members was experienced, the more students felt ridiculed, so there was a strong statistically significant association between the perceived pressure to be thin from family members and reporting feelings of being ridiculed, *χ*^2^(25) = 180.135, *φ* = 0.760, *p* < 0.0005.

The analysis of the students’ residency, current weight, presence of sleep difficulties, BMI and reporting the feeling of being ridiculed, did not reveal any statistically significant association between these parameters (*p* > 0.05). Regarding the personal medical history, 50.64% reported a family history of difficulties in maintaining a normal weight, 6.41% were diagnosed with chronic disease, and 39.74% had a family history of chronic diseases. More than half of the students with sleep problems reported a family history of difficulties in maintaining a normal weight, compared to only 43.7% of the students with normal sleep, the association between these variables being relatively weak but statistically significant, *χ*^2^(1) = 4.132, *φ* = 0.115, *p* = 0.042.

Students with a family history of difficulties in maintaining a normal weight experienced more pressure from family members and social pressure about being thin, compared to others, the differences being statistically significant (*χ*^2^(5) = 14.441, *φ* = 0.215, *p* = 0.013 for pressure from society, and *χ*^2^(5) = 11.730, *φ* = 0.194, *p* = 0.039 for pressure experienced from family members).

The existence of chronic diseases, both personally and within the family, is not associated with any of the previously presented parameters (*p* > 0.05). Regarding the existence of major events with negative impact, 10.26% never or only rarely experienced such events, while 17.63% felt them often, usually or always.

Within the group of students who often, usually, or always experienced major negative impact events, 26.4% of the students self-reported sleep difficulties, compared to only 5.0% of the students who had normal sleep. Thus, there was a statistically significant moderate association between sleep problems and the presence of events with a negative impact, *χ*^2^(5) = 35.814, *φ* = 0.339, *p* < 0.0005.

Almost a quarter of students who always experienced pressure from family members regarding their weight (23.3%) usually or always experienced events with a major negative impact. The percentage decreases to approximately 6% as the pressure felt from family members was less, thus identifying a moderately strong statistically significant association between family pressure and the presence of major events with negative impact, *χ*^2^(25) = 73.306, *φ* = 0.485, *p* < 0.0005.

Regarding the BMI category, obese students declared the occurrence of events with major negative impact only rarely or at most sometimes, while 22.7% of overweight students experience these events often or usually. This percentage decreases for students with a normal weight or underweight students, being around 11%. These differences are statistically significant, thus there is a significant moderate association between BMI and the presence of events with negative impact, *χ*^2^(15) = 37.333, *φ* = 0.346, *p* = 0.001. The analysis of students regarding gender and residency and the existence of major events with negative impact did not reveal any statistically significant association between these variables (*p* > 0.05).

### 3.3 Perception of social pressure

Regarding the pressure to be thin from family members, 34.29% never or only rarely experienced such pressure, while 25.01% were often, usually or always under this pressure.

Within the group of students who often, usually, or always felt pressure to be thin from family members, 33.3% of students reported the presence of sleep difficulties, compared to only 12.8% of students who had normal sleep. Thus, there was a statistically significant moderate association between sleep problems and family pressure, *χ*^2^(5) = 21.332, *φ* = 0.261, *p* = 0.001.

More than half of obese students (54.5%) and 15.1% of overweight students always felt pressure from family members. High percentages were also identified for pressure felt often or usually, from 37.4% of obese students and 24.5% of overweight students. Values were reversed for normal weight and underweight students, with a moderately strong significant association between BMI and perceived family pressure, *χ*^2^(15) = 65.966, *φ* = 0.460, *p* < 0.0005.

The analysis of students regarding gender and residency, as well as the existence of major events with negative impact, did not reveal any statistically significant association between these variables (*p* > 0.05).

Also, 32.37% never experienced social pressure to be thin, and 30.13% were often, usually or always exposed to this pressure.

The association between gender and social pressure to be thin was statistically significant, *χ*^2^(5) = 16.410, *φ* = 0.229, *p* = 0.006, 33.6% of females often, usually or always feeling this pressure, compared to only 17.6% among males. Social pressure was felt significantly more by females than by males.

Also, almost half of obese students (45.5%) always experienced social pressure, as do 17.0% of overweight students, the percentages being much lower for the other two categories. Thus, the association between BMI and social pressure was moderate and statistically significant, *χ*^2^(15) = 26.334, *φ* = 0.291, *p* = 0.035.

Therefore, the acquired data indicate that respondents experienced a wide range of health and psychological wellbeing issues, as well as significant social pressure related to physical appearance. These results emphasize the need for targeted interventions to improve health status and the quality of life among students attending medical schools.

### 3.4 EDI-3 results

To identify variables that had a significant impact on the likelihood that students were at risk of developing ED, the following data were used as inputs into a binomial logistic regression model: gender, age, residency, marital status, college, BMI value, sleep difficulties, reporting the presence of negative events, pressure to be thin from family members and society, reporting feelings of being ridiculed, family history of chronic diseases, family history of mental illness, family history of difficulties in maintaining a normal weight. The linearity of the continuous variables in relation to the logit of the dependent variable was assessed by the Box-Tidwell procedure. A Bonferroni correction was applied using the 22 terms in the model, resulting in a statistical significance threshold of *p* < 0.002273. Based on this evaluation, all continuous independent variables were found to be linearly related to the logit of the dependent variable. There was only one standardized residual value approximately equal to 3 × standard deviation (3.104), which was maintained in the analysis. The logistic regression model was statistically significant, *χ*^2^(14) = 142.209, *p* < 0.0005. The model explained 50.3% of the variation in risk of developing ED and correctly classified 78.7% of cases. The sensitivity was 62.3%, the specificity was 88.3%, the positive predictive value was 76.04%, and the negative predictive value was 80.09%. The area under the ROC curve was 0.867 (95% CI, 0.827 to 0.907), indicating an excellent level of discrimination. Of the 14 predictor variables, only five were statistically significant: gender, BMI value, sleep difficulties, family pressure and reporting the feeling of being ridiculed (Table 5).

**Table 5:** Binomial regression results.

Parameter	*p*	Odds ratio^*^	Range of confidence 95%	
			Minimum	Maximum
Gender	0.002	4.183	1.699	10.303
Age	0.339	0.948	0.849	1.058
Residence	0.365	0.713	0.343	1.482
Marital status	0.075	1.799	0.943	3.432
Faculty	0.327	0.819	0.550	1.220
BMI	<0.0005	1.253	1.131	1.388
Sleep	0.005	2.712	1.345	5.468
Major events with negative impact	0.723	1.060	0.767	1.467
Pressure to be thin from family	0.011	1.417	1.084	1.853
Pressure to be thin from society/media	0.252	1.164	0.898	1.508
Weight/appearance ridicule	0.044	1.306	1.008	1.692
Family history of chronic diseases	0.191	1.562	0.800	3.051
Family history of mental diseases	0.538	0.748	0.297	1.885
Family history of difficulty maintaining a normal wight/ED	0.447	1.279	0.678	2.415

Females were 4.183 times more likely to be at increased risk of developing ED compared to males. Students who experienced sleep difficulties were 2.712 times more likely to have an increased risk of ED. Increased family pressure to be thin, increased BMI, and more frequent feeling of being ridiculed were associated with an increased risk of ED.

The scale “Drive for thinness” (DT) comprised values from 0 to 28, with a mean of 9.21 ± 8.587. Based on these values, the clinical significance of the computed values was determined. The distribution of the study group according to the parameters under study and the clinical significance is highlighted in Table 6. Statistically significant differences were observed relative to gender, 9.4% of females having values with high significance, compared to 0% in the case of males (*p* = 0.001).

**Table 6:** Distribution of the study group relative to the scale “Drive for thinness” (DT).

Parameter	Category	Clinical significance			*p*
		Low	Standard	High	
	TOTAL	211 (67.63%)	78 (25.00%)	23 (7.37%)	–
Gender	M	58 (85.3%)	10 (14.7%)	0 (0%)	0.001
	F	153 (62.7%)	68 (27.9%)	23 (9.4%)	
Age	<20 years old	124 (67.4%)	45 (24.5%)	15 (8.2%)	0.808
	≥20 years old	87 (68%)	33 (25.8%)	8 (6.3%)	
Faculty	General Medicine	120 (66.7%)	43 (23.9%)	17 (9.4%)	0.233
	Dental Medicine	37 (75.5%)	9 (18.4%)	3 (6.1%)	
	Midwives and Nurses	54 (65.1%)	26 (31.3%)	3 (3.6%)	
Year of study	1	162 (67.5%)	61 (25.4%)	17 (7.1%)	0.261
	2	24 (64.9%)	11 (29.7%)	2 (5.4%)	
	3	11 (55%)	5 (25%)	4 (20%)	
	4	12 (92.3%)	1 (7.7%)	0 (0%)	
	5	2 (100%)	0 (0%)	0 (0%)	
Marital status	Single	119 (65.7%)	48 (26.5%)	14 (7.7%)	0.704
	In a relationship	92 (70.2%)	30 (22.9%)	9 (6.9%)	
Residence	Urban	155 (67.4%)	57 (24.8%)	18 (7.8%)	0.874
	Rural	56 (68.3%)	21 (25.6%)	5 (6.1%)	
Perceived income level	Low	28 (66.7%)	13 (31%)	1 (2.4%)	0.319
	Average	167 (69%)	57 (23.6%)	18 (7.4%)	
	High	16 (57.1%)	8 (28.6%)	4 (14.3%)	
Self-reported sleep difficulties in the last 6 months^*^	Yes	110 (59.1%)	55 (29.6%)	21 (11.3%)	<0.0005
	No	101 (80.2%)	23 (18.3%)	2 (1.6%)	
Weight/appearance ridicule	Never	71 (84.5%)	9 (10.7%)	4 (4.8%)	<0.0005
	Rarely	69 (82.1%)	12 (14.3%)	3 (3.6%)	
	Sometimes	42 (67.7%)	17 (27.4%)	3 (4.8%)	
	Often	16 (50.0%)	15 (46.9%)	1 (3.1%)	
	Usually	5 (31.3%)	9 (56.3%)	2 (12.5%)	
	Always	8 (23.5%)	16 (47.1%)	10 (29.4%)	
Pressure to be thin from family	Never	100 (93.5%)	6 (5.6%)	1 (0.9%)	<0.0005
	Rarely	42 (66.7%)	17 (27.0%)	4 (6.3%)	
	Sometimes	41 (64.1%)	18 (28.1%)	5 (7.8%)	
	Often	17 (56.7%)	13 (43.3%)	0 (0%)	
	Usually	6 (33.3%)	10 (55.6%)	2 (11.1%)	
	Always	5 (16.7%)	14 (46.7%)	11 (36.7%)	
Pressure to be thin from media	Never	89 (88.1%)	10 (9.9%)	2 (2.0%)	<0.0005
	Rarely	51 (85.0%)	9 (15.0%)	0 (0%)	
	Sometimes	33 (57.9%)	18 (31.6%)	6 (10.5%)	
	Often	20 (58.8%)	14 (41.2%)	0 (0%)	
	Usually	8 (38.1%)	12 (57.1%)	1 (4.8%)	
	Always	10 (25.6%)	15 (38.5%)	14 (35.9%)	
Family history of difficulty maintaining a normal weight/ED	Yes	98 (62%)	40 (25.3%)	20 (12.7%)	0.010
	No	113 (73.4%)	38 (24.7%)	3 (1.9%)	
Personal history of chronic disease	Yes	14 (70.0%)	5 (25.0%)	1 (5.0%)	0.914
	No	197 (67.5%)	73 (25.0%)	22 (7.5%)	
Family history of chronic disease	Yes	82 (66.1%)	30 (24.2%)	12 (9.7%)	0.448
	No	129 (68.6%)	48 (25.5%)	11 (5.9%)	
Family history of mental disease	Yes	25 (58.1%)	15 (34.9%)	3 (7%)	0.269
	No	186 (69.1%)	63 (23.4%)	20 (7.4%)	
Major events with negative impact	Never	24 (75.0%)	8 (25.0%)	0 (0%)	0.008
	Rarely	85 (78.0%)	21 (19.3%)	3 (2.8%)	
	Sometimes	76 (65.5%)	28 (24.1%)	12 (10.3%)	
	Often	19 (50%)	14 (36.8%)	5 (13.2%)	
	Usually	5 (35.7%)	6 (42.9%)	3 (21.4%)	
	Always	2 (66.7%)	1 (33.3%)	0 (0%)	
BMI	Underweight	42 (93.3%)	2 (4.4%)	1 (2.2%)	<0.0005
	Normal weight	139 (68.5%)	50 (24.6%)	14 (6.9%)	
	Overweight	26 (49.1%)	20 (37.7%)	7 (13.2%)	
	Obese	4 (36.4%)	6 (54.5%)	1 (9.1%)	

* Self-reported sleep difficulties in the last 6 months.

Similarly, statistically significant differences were observed relative to the presence of sleep difficulties, with 11.3% of students who reported such problems having values of high significance, compared to only 1.6% of students who reported no sleep issues. The pressure of being thin, experienced both from family members and from society, as well as the feeling of being ridiculed, were statistically significantly associated with the level of clinical significance of the DT scale, as follows: the more pressure or ridiculed students felt, the higher the levels of the DT scale. Similar results were also obtained for reporting of major events with a negative impact, this issue being associated with the clinical level of the scale (*p* = 0.008). Family history relative to difficulties in maintaining a normal weight shows statistically significant differences between the levels of clinical significance of the DT scale: 12.7% of students with a family history had a high level, compared to only 1.9% of students who did not report such a family history. Last but not least, the BMI category reflected the increased desire of overweight and obese students to be thin (having high clinically meaningful scale values) compared to underweight students or students with a normal weight.

The Scale “Bulimia” (B) comprised values between 0 and 32, with a mean of 4.73 ± 6.251. Based on these values, the clinical significance of the computed values was determined. The distribution of the study group according to the studied parameters and the clinical significance is highlighted in Table 7. The level of clinical significance of the scale B was statistically significantly associated with a number of variables previously identified as having statistical significance for the DT scale as well: sleep difficulties (where 15.6% of students with sleep problems present a high level of the B scale), the feeling of being ridiculed, the pressure of being thin experienced both from family members and from society, the existence of events with a major negative impact, BMI category, family history of ED; the higher the values of these variables, the higher the level of clinical significance of the scale (*p* < 0.05).

**Table 7:** Distribution of the study group relative to the scale “Bulimia” (B).

Parameter	Category	Clinical significance			*p*
		Low	Standard	High	
	TOTAL	165 (52.88%)	113 (36.22%)	34 (10.90%)	–
Gender	M	39 (57.4%)	26 (38.2%)	3 (4.4%)	0.151
	F	126 (51.6%)	87 (35.7%)	31 (12.7%)	
Age	<20 years old	95 (51.6%)	68 (37%)	21 (11.4%)	0.856
	≥20 years old	70 (54.7%)	45 (35.2%)	13 (10.2%)	
Faculty	General Medicine	91 (50.6%)	66 (36.7%)	23 (12.8%)	0.503
	Dental Medicine	25 (51%)	18 (36.7%)	6 (12.2%)	
	Midwives and Nurses	49 (59%)	29 (34.9%)	5 (6%)	
Year of study	1	128 (53.3%)	88 (36.7%)	24 (10%)	0.435
	2	18 (48.6%)	15 (40.5%)	4 (10.8%)	
	3	8 (40%)	7 (35%)	5 (25%)	
	4	9 (69.2%)	3 (23.1%)	1 (7.7%)	
	5	2 (100%)	0 (0%)	0 (0%)	
Marital status	Single	86 (47.5%)	71 (39.2%)	24 (13.3%)	0.060
	In a relationship	79 (60.3%)	42 (32.1%)	10 (7.6%)	
Residence	Urban	119 (51.7%)	84 (36.5%)	27 (11.7%)	0.668
	Rural	46 (56.1%)	29 (35.4%)	7 (8.5%)	
Perceived income level	Low	23 (54.8%)	13 (31%)	6 (14.3%)	0.344
	Average	132 (54.5%)	86 (35.5%)	24 (9.9%)	
	High	10 (35.7%)	14 (50%)	4 (14.3%)	
Self-reported sleep difficulties in the last 6 months^*^	Yes	87 (46.8%)	70 (37.6%)	29 (15.6%)	0.002
	No	78 (61.9%)	43 (34.1%)	5 (4.0%)	
Weight/appearance ridicule	Never	57 (67.9%)	25 (29.8%)	2 (2.4%)	<0.0005
	Rarely	48 (57.1%)	30 (35.7%)	6 (7.1%)	
	Sometimes	29 (46.8%)	27 (43.5%)	6 (9.7%)	
	Often	15 (46.9%)	13 (40.6%)	4 (12.5%)	
	Usually	4 (25.0%)	6 (37.5%)	6 (37.5%)	
	Always	12 (35.3%)	12 (35.3%)	10 (29.4%)	
Pressure to be thin from family	Never	71 (66.4%)	35 (32.7%)	1 (0.9%)	<0.0005
	Rarely	38 (60.3%)	23 (36.5%)	2 (3.2%)	
	Sometimes	29 (45.3%)	25 (39.1%)	10 (15.6%)	
	Often	14 (46.7%)	13 (43.3%)	3 (10.0%)	
	Usually	6 (33.3%)	5 (27.8%)	7 (38.9%)	
	Always	7 (23.3%)	12 (40%)	11 (36.7%)	
Pressure to be thin from media	Never	67 (66.3%)	31 (30.7%)	3 (3.0%)	<0.0005
	Rarely	36 (60.0%)	22 (36.7%)	2 (3.3%)	
	Sometimes	27 (47.4%)	22 (38.6%)	8 (14.0%)	
	Often	16 (47.1%)	12 (35.3%)	6 (17.6%)	
	Usually	7 (33.3%)	11 (52.4%)	3 (14.3%)	
	Always	12 (30.8%)	15 (38.5%)	12 (30.8%)	
Family history of difficulty maintaining a normal weight/ED	Yes	75 (47.5%)	60 (38%)	23 (14.6%)	0.050
	No	90 (58.4%)	53 (34.4%)	11 (7.1%)	
Personal history of chronic disease	Yes	10 (50%)	8 (40%)	2 (10%)	0.935
	No	155 (53.1%)	105 (36%)	32 (11%)	
Family history of chronic disease	Yes	63 (50.8%)	48 (38.7%)	13 (10.5%)	0.758
	No	102 (54.3%)	65 (34.6%)	21 (11.2%)	
Family history of mental disease	Yes	18 (41.9%)	15 (34.9%)	10 (23.3%)	0.017
	No	147 (54.6%)	98 (36.4%)	24 (8.9%)	
Major events with negative impact	Never	19 (59.4%)	10 (31.3%)	3 (9.4%)	0.001
	Rarely	69 (63.3%)	37 (33.9%)	3 (2.8%)	
	Sometimes	57 (49.1%)	45 (38.8%)	14 (12.1%)	
	Often	12 (31.6%)	15 (39.5%)	11 (28.9%)	
	Usually	5 (35.7%)	6 (42.9%)	3 (21.4%)	
	Always	3 (100%)	0 (0%)	0 (0%)	
BMI	Underweight	28 (62.2%)	16 (35.6%)	1 (2.2%)	0.002
	Normal weight	113 (55.7%)	72 (35.5%)	18 (8.9%)	
	Overweight	22 (41.5%)	20 (37.7%)	11 (20.8%)	
	Obese	2 (18.2%)	5 (45.5%)	4 (36.4%)	

* Self-reported sleep difficulties in the last 6 months.

In contrast to the DT scale, family history of mental illness was associated with B scale values, as follows: 23.3% of students who reported such a history had a high level of clinical significance of the B scale, compared to 8.9% of students without a similar history.

The scale Body dissatisfaction (BD) comprised values between 0 and 40, with a mean of 12.93 ± 10.075. Based on these values, the clinical significance of the computed values was determined. The distribution of the study group according to the studied parameters and the clinical significance is highlighted in Table 8. In comparison with DT and B scales, the presence of major negative events is no longer significantly statistically associated with body dissatisfaction (*p* = 0.063). Also, the majority of obese students (63.6%) showed a high level of body dissatisfaction relative to their own bodies, in comparison with only 9.1% who feel the drive for thinness.

**Table 8:** Distribution of the study group relative to the scale “Body dissatisfaction” (BD).

Parameter	Category	Clinical significance			*p*
		Low	Standard	High	
	TOTAL	148 (47.44%)	135 (43.27%)	29 (9.29%)	–
Gender	M	41 (60.3%)	25 (36.8%)	2 (2.9%)	0.022
	F	107 (43.9%)	110 (45.1%)	27 (11.1%)	
Age	<20 years old	85 (46.2%)	85 (46.2%)	14 (7.6%)	0.300
	≥20 years old	63 (49.2%)	50 (39.1%)	15 (11.7%)	
Faculty	General Medicine	87 (48.3%)	74 (41.1%)	19 (10.6%)	0.255
	Dental Medicine	27 (55.1%)	21 (42.9%)	1 (2%)	
	Midwives and Nurses	34 (41%)	40 (48.2%)	9 (10.8%)	
Year of study	1	117 (48.8%)	105 (43.8%)	18 (7.5%)	0.258
	2	15 (40.5%)	14 (37.8%)	8 (21.6%)	
	3	8 (40%)	9 (45%)	3 (15%)	
	4	7 (53.8%)	6 (46.2%)	0 (0%)	
	5	1 (50%)	1 (50%)	0 (0%)	
Marital status	Single	80 (44.2%)	84 (46.4%)	17 (9.4%)	0.379
	In a relationship	68 (51.9%)	51 (38.9%)	12 (9.2%)	
Residence	Urban	110 (47.8%)	101 (43.9%)	19 (8.3%)	0.572
	Rural	38 (46.3%)	34 (41.5%)	10 (12.2%)	
Perceived income level	Low	19 (45.2%)	18 (42.9%)	5 (11.9%)	0.824
	Average	116 (47.9%)	106 (43.8%)	20 (8.3%)	
	High	13 (46.4%)	11 (39.3%)	4 (14.3%)	
Self-reported sleep difficulties in the last 6 months^*^	Yes	75 (40.3%)	88 (47.3%)	23 (12.4%)	0.003
	No	73 (57.9%)	47 (37.3%)	6 (4.8%)	
Weight/appearance ridicule	Never	62 (73.8%)	20 (23.8%)	2 (2.4%)	<0.0005
	Rarely	49 (58.3%)	33 (39.3%)	2 (2.4%)	
	Sometimes	22 (35.5%)	35 (56.5%)	5 (8.1%)	
	Often	9 (28.1%)	20 (62.5%)	3 (9.4%)	
	Usually	3 (18.8%)	9 (56.2%)	4 (25.0%)	
	Always	3 (8.8%)	18 (52.9%)	13 (38.2%)	
Pressure to be thin from family	Never	71 (66.4%)	36 (33.6%)	0 (0%)	<0.0005
	Rarely	35 (55.6%)	27 (42.9%)	1 (1.6%)	
	Sometimes	26 (40.6%)	32 (50%)	6 (9.4%)	
	Often	7 (23.3%)	20 (66.7%)	3 (10.0%)	
	Usually	7 (38.9%)	6 (33.3%)	5 (27.8%)	
	Always	2 (6.7%)	14 (46.7%)	14 (46.7%)	
Pressure to be thin from media	Never	67 (66.3%)	32 (31.7%)	2 (2%)	<0.0005
	Rarely	37 (61.7%)	21 (35%)	2 (3.3%)	
	Sometimes	23 (40.4%)	26 (45.6%)	8 (14.0%)	
	Often	8 (23.5%)	24 (70.6%)	2 (5.9%)	
	Usually	5 (23.8%)	13 (61.9%)	3 (14.3%)	
	Always	8 (20.5%)	19 (48.7%)	12 (30.8%)	
Family history of difficulty maintaining a normal weight/ED	Yes	70 (44.3%)	64 (40.5%)	24 (15.2%)	0.001
	No	78 (50.6%)	71 (46.1%)	5 (3.2%)	
Personal history of chronic disease	Yes	11 (55.0%)	8 (40.0%)	1 (5.0%)	0.693
	No	137 (46.9%)	127 (43.5%)	28 (9.6%)	
Family history of chronic disease	Yes	59 (47.6%)	52 (41.9%)	13 (10.5%)	0.819
	No	89 (47.3%)	83 (44.1%)	16 (8.5%)	
Family history of mental disease	Yes	19 (44.2%)	17 (39.5%)	7 (16.3%)	0.236
	No	129 (48.0%)	118 (43.9%)	22 (8.2%)	
Major events with negative impact	Never	19 (59.4%)	12 (37.5%)	1 (3.1%)	0.063
	Rarely	60 (55.0%)	44 (40.4%)	5 (4.6%)	
	Sometimes	51 (44.0%)	50 (43.1%)	15 (12.9%)	
	Often	15 (39.5%)	18 (47.4%)	5 (13.2%)	
	Usually	2 (14.3%)	9 (64.3%)	3 (21.4%)	
	Always	1 (33.3%)	2 (66.7%)	0 (0%)	
BMI	Underweight	29 (64.4%)	16 (35.6%)	0 (0%)	<0.0005
	Normal weight	107 (52.7%)	83 (40.9%)	13 (6.4%)	
	Overweight	11 (20.8%)	33 (62.3%)	9 (17.0%)	
	Obese	1 (9.1%)	3 (27.3%)	7 (63.6%)	

* Self-reported sleep difficulties in the last 6 months.

The composite scale ECC (Eating Concerns Composite) is computed based on the values of the scales DT, B and BD. This comprised values between 0 and 86, with a mean of 26.87 ± 21.210. Based on these values, the clinical significance of the computed values was determined. The distribution of the study group according to the studied parameters and the clinical significance is highlighted in Table 9. Because it is a composite scale based on simple scales DT, B and BD presented before, its levels of clinical significance are significatively statistically associated with the same variables identified for simple scales.

**Table 9:** Distribution of the study group relative to the composite scale ECC and the parameters included in the study.

Parameter	Category	Clinical significance			*p*
		Low	Standard	High	
	TOTAL	196 (62.9%)	75 (24.0%)	41 (13.1%)	–
Gender	M	52 (76.5%)	14 (20.6%)	2 (2.9%)	0.007
	F	144 (59.0%)	61 (25.0%)	39 (16.0%)	
Age	<20 years old	115 (62.5%)	42 (22.8%)	27 (14.7%)	0.582
	≥20 years old	81 (63.3%)	33 (25.8%)	14 (10.9%)	
Faculty	General Medicine	197 (59.4%)	45 (25.0%)	28 (15.6%)	0.321
	Dental Medicine	35 (71.4%)	8 (16.3%)	6 (12.2%)	
	Midwives and Nurses	54 (65.1%)	22 (26.5%)	7 (8.4%)	
Year of study	1	153 (63.7%)	57 (23.8%)	30 (12.5%)	0.567
	2	21 (56.8%)	10 (27.0%)	6 (16.2%)	
	3	10 (50.0%)	5 (25.0%)	5 (25.0%)	
	4	10 (76.9%)	3 (23.1%)	0 (0.0%)	
	5	2 (100%)	0 (0.0%)	0 (0.0%)	
Marital status	Single	110 (60.8%)	45 (24.9%)	26 (14.4%)	0.638
	In a relationship	86 (65.6%)	30 (22.9%)	15 (11.5%)	
Residence	Urban	141 (61.3%)	56 (24.3%)	33 (14.3%)	0.516
	Rural	55 (67.1%)	19 (23.2%)	8 (9.8%)	
Perceived income level	Low	24 (57.1%)	11 (26.2%)	7 (16.7%)	0.791
	Average	156 (64.5%)	57 (23.6%)	29 (12.0%)	
	High	16 (57.1%)	7 (25.0%)	5 (17.9%)	
Self-reported sleep difficulties in the last 6 months^*^	Yes	100 (53.8%)	54 (29.0%)	32 (17.2%)	<0.0005
	No	96 (76.2%)	21 (16.7%)	9 (7.1%)	
Weight/appearance ridicule	Never	71 (84.5%)	10 (11.9%)	3 (3.6%)	<0.0005
	Rarely	61 (72.6%)	19 (22.6%)	4 (4.8%)	
	Sometimes	36 (58.1%)	19 (30.6%)	7 (11.3%)	
	Often	16 (50.0%)	13 (40.6%)	3 (9.4%)	
	Usually	3 (18.8%)	7 (43.8%)	6 (37.5%)	
	Always	9 (26.5%)	7 (20.6%)	18 (52.9%)	
Pressure to be thin from family	Never	94 (87.9%)	12 (11.2%)	1 (0.9%)	<0.0005
	Rarely	45 (71.4%)	12 (19.0%)	6 (9.5%)	
	Sometimes	35 (54.7%)	22 (34.4%)	7 (10.9%)	
	Often	13 (43.3%)	14 (46.7%)	3 (10.0%)	
	Usually	5 (27.8%)	7 (38.9%)	6 (33.3%)	
	Always	4 (13.3%)	8 (26.7%)	18 (60.0%)	
Pressure to be thin from media	Never	86 (85.1%)	14 (13.9%)	1 (1.0%)	<0.0005
	Rarely	47 (78.3%)	12 (20.0%)	1 (1.7%)	
	Sometimes	30 (52.6%)	18 (31.6%)	9 (15.8%)	
	Often	17 (50.0%)	12 (35.3%)	5 (14.7%)	
	Usually	8 (38.1%)	9 (42.9%)	4 (19.0%)	
	Always	8 (20.5%)	10 (25.6%)	21 (53.8%)	
Family history of difficulty maintaining a normal weight/ED	Yes	87 (55.1%)	39 (24.7%)	32 (20.3%)	<0.0005
	No	109 (70.8%)	36 (23.4%)	9 (5.8%)	
Personal history of chronic disease	Yes	11 (55.0%)	8 (40.0%)	1 (5.0%)	0.169
	No	185 (63.4%)	67 (22.9%)	40 (13.7%)	
Family history of chronic disease	Yes	71 (57.3%)	34 (27.4%)	19 (15.3%)	0.254
	No	125 (66.5%)	41 (21.8%)	22 (11.7%)	
Family history of mental disease	Yes	22 (51.2%)	11 (25.6%)	10 (23.3%)	0.082
	No	174 (64.7%)	64 (23.8%)	31 (11.5%)	
Major events with negative impact	Never	25 (78.1%)	6 (18.8%)	1 (3.1%)	<0.0005
	Rarely	81 (74.3%)	22 (20.2%)	6 (5.5%)	
	Sometimes	69 (59.5%)	29 (25.0%)	18 (15.5%)	
	Often	13 (34.2%)	13 (34.2%)	12 (31.6%)	
	Usually	6 (42.9%)	4 (28.6%)	4 (28.6%)	
	Always	2 (66.7%)	1 (33.3%)	0 (0.0%)	
BMI	Underweight	42 (93.3%)	3 (6.7%)	0 (0.0%)	<0.0005
	Normal weight	129 (63.5%)	52 (25.6%)	22 (10.8%)	
	Overweight	23 (43.4%)	17 (32.1%)	13 (24.5%)	
	Obese	2 (18.2%)	3 (27.3%)	6 (54.5%)	

* Self-reported sleep difficulties in the last 6 months.

The BMI index was represented in the study both as categorical values (underweight, normal weight, overweight and obese—categories analyzed in Tables 6–9) and as numerical values (between 15.62 and 39.79).

The analysis of numerical values of this parameter did not reveal a monotonic relationship between the scores of the DT, B, ECC and BMI scales, therefore these variables were transformed by applying a log10 transformation. A subsequent statistical analysis was made with the transformed parameters. A Spearman correlation was performed to evaluate the connection between the BD scale regarding the appearance and the BMI index dissatisfaction.

The preliminary analysis showed that the relationship was monotonic, as assessed by visual inspection of the graph. There was a statistically significant moderate positive correlation between the BD values and BMI indices, rs(310) = 0.371, *p* < 0.0005. Thus, an increase of the BMI index was moderately associated with an increase of the BD value. Similar analyzes were performed for the transformed variables corresponding to the scores of the DT, B and ECC scales. For all three, there was a statistically significant moderate positive correlation with the BMI indices, rs(310) = 0.243, *p* < 0.0005 for EDI3_B, rs(310) = 0.323, *p* < 0.0005 for EDI3_DT and rs(310) = 0.389, *p* < 0.0005 for ECC. Thus, an increase of the BMI index was moderately associated with an increase of these scores.

### 3.5 Psychological scales results

The analysis of the composite psychological scales presented in the EDI questionnaire showed that their levels of clinical significance are statistically significantly associated with most of the statistically significant variables that were previously identified in the study, and described for the DT, B, BD and ECC scales.

In contrast to these scales, the composite scale SIC (Ineffectiveness) is also significantly associated with the income reported by students, as follows: 28.6% of low-income students and 32.1% of high-income students have a high level of the scale, in comparison with only 10.3% of average-income students. Furthermore, the BMI category is not associated with the SIC scale.

Clinical significance levels of the composite scale IPC (reflecting interpersonal problems) were additionally associated with marital status, 21.5% of single students showing high scale scores, compared to only 14.5% of students in a relationship (*p* = 0.039). Students’ income also is statistically significant associated with the levels of the scale: as income increases, the number of students with high scores of IPC scale decreases. Also, the BMI category is not associated with the presence of interpersonal problems.

The APC scale (of affective problems) is associated in addition with the age category of the participants in the study: differences are not present at the high clinical level of the scores, but at the low level: 40.8% of students under 20 years old have a low score of the scale (included in the low clinical level), compared with 54.7% of students older than or equal to 20 years old (*p* = 0.039). Also, the clinical significance level of the APC scale is not associated with family history of eating disorders, or with the BMI category. Similarly, the simple ED scale (emotional dysregulation) is additionally associated with age category, but not with BMI categories.

The OC scale (reflecting overcontrol) does not show statistically significant differences of the clinical levels in relation to gender, income, family history of ED or BMI category of the participants, compared to ECC scale.

The GPM scale (reflecting psychological maladjustment), as well as the MF simple scale (Maturity Fears) do not show statistically significant differences of the clinical levels in relation to any variable in this study.

The simple scales LSE (low self-esteem), II (interpersonal insecurity), IA (interpersonal alienation), ID (interoceptive deficits), P (perfectionism) and A (ascetism) are basically associated with the same variables identified for the simple scales DT, B and BD.

The simple scale PA (personal alienation) was statistically significant associated in addition to the other scales, with the faculty attended by the students (18.9% of the students from the General Medicine specialization showed an increased level of clinical significance of the scale values, compared to 16.3% of the students from Dental Medicine, and 8.4% of the students from the Faculty of Midwives and Nurses), with background of origin (18.7% of urban students have a high level, compared to 7.3% of rural student), with income (approximately a quarter of low- and high-income students shows a high clinical level, compared to 12.8% of average-income students), with personal history of chronic disease, but also with family history of mental illness. Also, the simple PA scale is not associated with BMI categories.

### 3.6 Comparison between medical students and non-clinical nationally representative sample of adults

The participants in the sample for calibration included 304 women from the general population, aged between 18 and 55 years (*m* = 24.97, *σ* = 8.81) (Miclea et al., 2011). The participants in our study had scores (DT: 9.21 ± 8.58, B: 4.73 ± 6.25, BD: 12.93 ± 10.07) on the three ED risk scales that were significantly higher than the people from the nationally representative sample (DT: 7.09 ± 7.42, 2.55 ± 4.02, 10.32 ± 9.16). Also, the medical students recorded much higher scores than the general population on all psychological scales, as can be seen in Table 10 (Miclea et al., 2011).

**Table 10:** Comparison between ED risk in the general population and the medical students from the University for Medicine and Pharmacy in Craiova.

Scale	General population^*^	University for Medicine and Pharmacy Craiova
ECC		
DT	7.09 ± 7.42	9.21 ± 8.58
B	2.55 ± 4.02	4.73 ± 6.25
BD	10.32 ± 9.16	12.93 ± 10.07
Psychological scales		
LSE	3.90 ± 3.97	5.86 ± 5.88
PA	4.47 ± 3.89	6.81 ± 5.96
II	6.60 ± 5.31	9.61 ± 6.42
IA	6.57 ± 4.37	8.34 ± 4.58
ID	6.44 ± 5.26	11.09 ± 7.87
ED	4.96 ± 4.55	8.17 ± 6.22
P	9.88 ± 4.86	12.57 ± 5.32
A	6.30 ± 4.38	8.31 ± 5.67
MF	12.38 ± 6.04	15.08 ± 7.26

* Garner (2010).

Differences between genders for the EDI-3 scales were performed in reference (Miclea et al., 2011) by comparing the mean scores for all scales. Two samples were selected: one consisting of 32 women aged between 18 and 55 years (*m* = 27.62, *σ* = 12.81) and the second consisting of 29 men aged between 18 and 57 years (*m* = 27.24, *σ* = 12.61). Both samples have similar demographic and BMI characteristics. Next, we wanted to see if there were gender differences in our sample, and, in this sense, we compared the mean scores of EDI-3 scales. In this case, too, significantly higher differences emerged between women and men, for all EDI-3 scales (Table 11) (Miclea et al., 2011).

**Table 11:** Gender comparison for EDI-3 scales between the general population and the students from the University of Medicine and Pharmacy of Craiova.

Scale	General population*		University for Medicine and Pharmacy Craiova	
	Men	Women	Men	Women
ECC				
DT	2.66 ± 3.66	8.03 ± 7.54	5.52 ± 5.43	10.18 ± 9.02
B	2.04 ± 2.38	2.00 ± 3.18	3.36 ± 4.415	5.05 ± 6.61
BD	5.18 ± 6.13	10.29 ± 9.28	10.29 ± 9.48	13.65 ± 10.16
Psychological scales				
LSE	3.28 ± 4.85	2.97 ± 3.19	4.78 ± 5.48	6.16 ± 5.96
PA	3.55 ± 4.12	4.48 ± 3.45	4.78 ± 5.48	7.22 ± 6.09
II	7.07 ± 5.87	6.73 ± 4.95	8.77 ± 6.01	9.82 ± 6.51
IA	7.04 ± 4.48	7.13 ± 4.06	7.33 ± 3.65	8.61 ± 4.75
ID	4.79 ± 5.16	6.10 ± 4.34	7.50 ± 6.10	12.04 ± 8.03
ED	5.36 ± 5.83	6.03 ± 4.60	6.92 ± 5.42	8.50 ± 6.39
P	11.79 ± 5.34	10.42 ± 5.42	11.19 ± 5.38	12.93 ± 5.25
A	6.41 ± 3.66	6.81 ± 3.82	9.42 ± 4.97	7.96 ± 5.82
MF	10.62 ± 4.07	13.23 ± 5.81	14.57 ± 5.95	15.21 ± 7.59

*Garner (2010).

## 4 Discussion

Universities all over the world recognize the importance of medical students` mental health due to the increasing mental health issues reported in the literature because of the academic requirements, personal and societal stressors (Estrella-Proaño et al., 2022; Valladares-Garrido et al., 2023). Eating disorders are severe mental health conditions that can affect a medical students physical and psychological health, social relationships, and academic performances (Hoteit et al., 2022). The objectives of our study were to determine the prevalence of ED risk for medical students and the associated risk factors and to compare the results to the general population in Romania.

Our study indicated worrying results regarding the prevalence of ED in medical students, 37.1% of them having a high clinical risk of developing an eating disorder characterized by significant preoccupations with dieting, weight, drive for thinness, intense fear of gaining weight, tendency toward compulsive eating, body dissatisfaction. These data were similar to studies performed in Europe and worldwide. Thus, a study by Ladner J, performed in four European universities: Chisinau (Ch) in Republic of Moldova, Cluj-Napoca (CN) in Romania, Miskolc (Ms) in Hungary and Rouen (R) in France showed that the ED global prevalence was 23.8% (Ladner et al., 2019). Another study aimed to determine the prevalence of eating disorders among students in Hungary, Poland and Ukraine, and reported the following percentages: 21.0% in Hungary, 19.7% in Poland and 36.9% in Ukraine (Kiss-Tóth et al., 2018; Iorga et al., 2019; Halbeisen et al., 2022).

Also, the prevalence score is significantly higher than the general population in Romania for women and men. Two studies with smaller samples of Pharmacy (no = 91) and Dental Medicine (no = 81) students reported similar results. Female students from Dental Medicine had higher scores than women in the general population for the following scales: low self-esteem, personal alienation, interpersonal insecurity, and interpersonal alienation. Male students had high scores in six (drive for thinness, low self-esteem, personal alienation, personal insecurity, interpersonal alienation, maturity fears) of the 12 scales. For Pharmacy students, higher values than the general population were reported for all scales, in the case of females, and for males, the same, except for the scales of self-esteem, interpersonal insecurity, interpersonal alienation, ascetism, maturity fears (Mocanu, 2013; Hudson et al., 2007). Thus, prevention, monitoring ED risk, and early identification of medical students with ED risk are essential in this population. Medical universities, by implementing specific strategies (for example: stress management workshops, awareness campaigns, regular ED screening, etc.), can better respond to their student’s psychological needs and enhance their wellbeing.

Female medical students have a significantly higher ED risk than males, who, in our opinion, also have a non-negligible risk of 23%. The data are in agreement with previous discoveries (Jahrami et al., 2019; Jahrami et al., 2019; Bizri et al., 2020; Culbert et al., 2021; Memon et al., 2012; Tetley et al., 2014). Also, female medical students are more vulnerable to the societal influences to be thin, they reported more sleeping difficulties, family history of ED and feelings of being ridiculed related to physical appearance than men did. In other words, the more family pressure is felt, the more ridiculed students feel. Socio-cultural pressure is an important risk factor and our results highlight once again the importance of family support, of culture and education regarding healthy behaviors, in order to reduce, as much as possible, the risk of developing or maintaining an eating disorder (Mocanu, 2013; Joja, 2001; Túry et al., 2020). One explanation for our results would be that the females are more likely subject to socio-cultural pressure to be thin, especially after the Revolution of 1989, when major social changes occurred, implicitly regarding the image of women. Thus, women started to be more concerned with themselves, to adhere to modern socio-cultural models, “to be thin” means to be appreciated, and, at the same time, females’ interest for dieting programs increased as a result of movies/media, which promote the idea of being thin (Kovács Krizbai and Szabó, 2009; Joja et al., 2015; Túry et al., 2023; Petunova et al., 2022; Allison et al., 2016; Pope Jr et al., 1984; Stice et al., 2001), but also as a result of the differences regarding the biological factors involved (Culbert et al., 2021). The results of another study showed that both psychological and physical aspects of the life of medical students are deeply affected by social media, which often promotes unattainable body standards (Stice and Whitenton, 2002).

Also, females presented more than males a higher body dissatisfaction, but also ineffectiveness and affective problems. Other research recorded similar results. ED were associated with being a female and the drive for thinness, bulimia, body dissatisfaction, high level of perfectionism, interpersonal insecurity (discomfort, restlessness, reluctance in social situations), and interoceptive deficits (confusion associated with the ability to recognize and appropriate response to emotional states). Trying to meet the standards imposed by success and beauty, being dissatisfied with their body image, they try to strictly control their meals (Joja, 2001). Another study reported high levels of perfectionism, body dissatisfaction and ineffectiveness in medical students (Bould et al., 2015). Therefore, the medical universities should aim to increase the mental health support for both women and men, by promoting a healthy body image, through educational programs, courses on health and wellness, counseling services, etc. Almost half of medical students in our sample (47.12%) were at high risk of bulimia. In the literature we can find similar data regarding the prevalence of bulimia among medical student population indicating that bulimia has high prevalence, and that ED are not isolated mental health conditions but that it reflects wider societal trends. These data are valuable for the medical universities so that they can develop policies and strategies to prevent ED risk and/or avoid raising the rates of the ED prevalence (Azzouzi et al., 2019; Mocanu, 2013; Jahrami et al., 2020; Smith et al., 2017).

A high BMI increases the risk of ED, so the overweight people and the obese are at higher risk of developing an ED than the underweight people. It is interesting that over a quarter of the people with normal weight are also at high risk of ED. A possible explanation for the fact that the underweight people are less liable to ED than the overweight people could be found in the biopsychosocial approach of ED, i.e., eating disorders have many causes, genetic/biological liabilities, psychological (desired body image, etc.), social (pressure from family and society), and these explain why a quite large number of people with normal weight are at high risk of ED. Similar results were obtained by other studies (Jahrami et al., 2020).

Residence, marital status and income are not risk factors for ED. An explanation could be the fact that, nowadays, students have very easy access to technology and internet, in Romania the cost for these services can be relatively low, which facilitates the access of the Romanian people to Western culture, regardless of whether they live in the city, or in the village, and, implicitly, to Western body image that promotes unrealistic beauty standards. A study showed that socioeconomic status of the medical students is not associated with ED (Azzouzi et al., 2019).

The regression analysis model showed that gender, sleep difficulties in the past 6 months, family pressure to be thin, and the feeling of being ridiculed related to physical appearance are associated risk factors with eating disorders. Thus, females compared to males have a four times higher risk of developing eating disorders and those who reported sleep problems have a 2.7 higher ED risk. To our surprise, no association of ED and media pressure was observed. Although both types of pressure, family and social, can contribute to ED development, it seems that the impact of media pressure on the ED risk is insignificant when included with other factors in an ensemble. An explanation for this phenomenon could be the fact that the effects of social pressure seem to be more versatile and easier to manage than family pressure, which can have a greater and persistent impact on the personality of individuals, and, implicitly, on ED risk (Smith et al., 2017; Guo et al., 2022; Zhang et al., 2024). In a meta-analysis which contained articles from 13 countries, Jahrami H. showed that sleep problems are common among medical students: they sleep an average of 6.3 h/night, 55% reported a low quality of sleep, 31% accused excessive daytime sleepiness. Some differences between countries were obtained, and this suggests that cultural values, local conditions, and the environment all have an impact on sleep practices and attitudes (Jahrami et al., 2024). In Romania, a study conducted on 81 students in Dental Medicine reported that ED risk is more often in females than in males, they sleep an average of 6.34 h per night (Ghamri et al., 2022), and another one, with a sample of *n* = 91 Pharmacy students, reported that these sleep an average of 7 h/night (Mocanu, 2013).

Regarding the psychological factors related to ED risk, we have found some concerning results. Over a quarter of medical students have issues related to personal insecurity, inadequacy, ineffectiveness and lack of self-worth, characteristic of individuals with ED. Also, nearly half of medical students showed significant deficiencies at the level of self-concept (emotional emptiness and loneliness). The literature indicates that self-esteem is a very important factor in developing and persistence the eating disorders (Hsu et al., 2024). Approximately 70.02% of medical students reported feelings of discomfort, worry and reluctance in social interactions, while over half reported lack of trust in social relationships and they believe that others do not offer enough affection. Additionally, 53.52% describe their social relationships as tense and generally of being of poor quality. Our results also showed that half of the students do not have the ability to cope with uncomfortable emotional experiences, may they be positive or negative. So, it is possible that their lack of functional coping strategies in dealing with both positive and negative emotions makes them perceive their social relationships so challenging (Iorga et al., 2019). Furthermore, 73.4% of medical students reported significant or extreme problems of emotional instability, impulsivity, anger and self-destructive behaviors that can be associated with substance abuse, alcohol, drugs or both. Almost 80% of them have perfectionism tendencies, while 43.59% have tendency to ascetism. Almost all of them show maturity fears. In the literature we can find explanation that can determine this fear, i.e., difficult economic times, social pressure to stay young, and/or internal fears of taking on increased responsibility (Jahrami et al., 2024). Surprisingly, despite of all the above stressors, 99% of the medical students presented a low level of global distress, possibly more as a result of minimizing the psychological anxieties, rather than the absence of distress (Miclea et al., 2011). Medical students must cope with high demands during the faculty (e.g.: busy schedule, large volume of information to be memorized, etc.) and the lack of social skills for emotional regulation are major risk factors for development of ED in this population. By helping them improve their stress coping skills, it will positive impact their wellbeing also (Pope et al., 1984).

These findings emphasize the importance of addressing eating disorders in greater detail, with more participants in nationwide studies, especially given that the training of medical professionals involves many hours of study, practice, demanding exams, and learning a big volume of information in a relatively short time. Identifying the risk factors and associated comorbidities can contribute to the development of effective strategies for prevention and early intervention. Also, medical schools should develop and implement complex services for their students like individual/group counseling services, stress management workshops focused on resilience building and functional coping strategies. By helping them improve their social skills they, as future doctors, will be able to communicate more effectively with their patients, be more empathic, so overall they will be better prepared professionally (Stice et al., 2001).

### 4.1 Practical implications

Based on the findings of this study, we recommend that developing stress management skills and positive coping strategies is crucial for the overall wellbeing of these students. Additionally, implementing support programs that promote both physical and mental health among medical students is essential.

Furthermore, it is imperative to raise awareness within the academic community and among families regarding the challenges associated with body weight, the stigma surrounding it, and the societal pressures related to thinness. The results underscore the need for counseling and psychological support services at medical universities to help students navigate their challenges.

In summary, a comprehensive approach to addressing these issues could lead to significant improvements in the health and quality of life for medical students.

### 4.2 Strengths and limitations

Our study is innovative in that we took in consideration a wide range of risk factors associated with ED and we have demonstrated, to the best of our knowledge, for the first time among Romanian medical students, family pressure to maintain a thin body has a greater impact on developing ED than the pressure to be thin from the media/society. These findings hold noteworthy practical implications.

Nonetheless, our study has several limitations. It was conducted at a single university center in South-Western Romania, making it difficult to generalize the results to all medical students in the country. Furthermore, the sample predominantly consisted of first and second-year students, with a lack of participation from final-year students, and the overall sample size was relatively small. Also, we did not gather any data about the student’s accommodation (whether students lived in dorms, shared houses, or with family) and did not use a standardized questionnaire for sleeping difficulties so, our results should be interpreted taking this in consideration also.

Despite these limitations, we believe our research makes a substantial contribution to the understanding of eating disorders in Romania, providing valuable insights with practical applications and suggesting further research directions in this field. Furthermore, additional research is needed to thoroughly investigate the cultural and societal influences on eating disorders among medical students. A deeper understanding of these factors will help us have a more complex picture of how lifestyle choices and dietary habits contribute to the onset and/or persistence of eating disorders in this population and its effects on their academic performances and their wellbeing overall.

## 5 Conclusion

This study identified a high prevalence of ED among female medical students from UMFCv, exceeding the prevalence in the general Romanian population. Additionally, we identified several risk factors including sleep difficulties, feelings of ridicule, family and social pressure to maintain a thin body, family history of ED and a high BMI. Moreover, family pressure has a greater impact on developing ED than social pressure.

These results suggest the complexity of ED and the need for further research to explore cultural and societal influences in this population. Implementing university policies focused on prevention of ED is essential for the mental health of the future medical specialists By enhancing their wellbeing, in the long future, this will impact the health of their future patients also.

## Data Availability

The raw data supporting the conclusions of this article will be made available by the authors without undue reservation.

## References

[ref1] Allison K. C. Spaeth A. Hopkins C. M. (2016). Sleep and eating disorders. Curr. Psychiatry Rep. 18:18. doi: 10.1007/s11920-016-0728-8 27553980

[ref2] American Psychiatric Association (2016). “Eating disorders” in DSM-5: diagnostic and statistical manual of mental disorders. 5th ed (Bucharest, Romania: Callisto Medical Publishing House), 329–354.

[ref3] Arcelus J. Mitchell A. J. Wales J. Nielsen S. (2011). Mortality rates in patients with anorexia nervosa and other eating disorders: a meta-analysis of 36 studies. Arch. Gen. Psychiatry 68, 724–731. doi: 10.1001/archgenpsychiatry.2011.74 21727255

[ref4] Azzouzi N. Ahid S. Bragazzi N. L. Berhili N. Aarab C. Aalouane R.. (2019). Eating disorders among Moroccan medical students: cognition and behavior. Psychol Res Behav Manag 12, 129–135. doi: 10.2147/PRBM.S165114 30881156 PMC6417001

[ref5] Baker J. H. Maes H. H. Lissner L. Aggen S. H. Lichtenstein P. Kendler K. S. (2009). Genetic risk factors for disordered eating in adolescent males and females. J. Abnorm. Psychol. 118, 576–586. doi: 10.1037/a0016314, PMID: 19685954 PMC4045449

[ref6] Barakat S. McLean S. A. Bryant E. Le A. Marks P. National Eating Disorder Research Consortium. (2023). Risk factors for eating disorders: findings from a rapid review. J. Eat. Disord. 11:8. doi: 10.1186/s40337-022-00717-4 36650572 PMC9847054

[ref9001] Bartuc M. Giosan C. Szentagotai A. Stefan S. Dobrean A. Păsărelu C.. (2023). Sănătatea mintală în funcție de stadiile de viață: afecțiuni tipice, riscuri și prevalențe la diverse categorii de vârstă în Tabloul sănătății mintale în România. Raportul studiului realizat de Consiliul Economic și Social.

[ref7] Bizri M. Geagea L. Kobeissy F. Talih F. (2020). Prevalence of eating disorders among medical students in a Lebanese medical school: a cross-sectional study. Neuropsychiatr. Dis. Treat. 16, 1879–1887. doi: 10.2147/NDT.S266241, PMID: 32801721 PMC7414930

[ref8] Bould H. Sovio U. Koupil I. Dalman C. Micali N. Lewis G.. (2015). Do eating disorders in parents predict eating disorders in children? Evidence from a Swedish cohort. Acta PsychiatrScand 132, 51–59. doi: 10.1111/acps.12389, PMID: 25572654

[ref9] Brumboiu M. I. Cazacu I. Zunquin G. Manole F. Mogosan C. I. Porrovecchio A.. (2018). Nutritional status and eating disorders among medical students from the Cluj-Napoca university center. Clujul. Med. 91, 414–421. doi: 10.15386/cjmed-1018. Epub 2018 Oct 30, PMID: 30564017 PMC6296733

[ref10] Cena H. Vandoni M. Magenes V. C. Di Napoli I. Marin L. Baldassarre P.. (2022). Benefits of exercise in multidisciplinary treatment of binge eating disorder in adolescents with obesity. Int. J. Environ. Res. Public Health 19:8300. doi: 10.3390/ijerph19148300, PMID: 35886152 PMC9315465

[ref11] Cepeda-Benito A. Moreno-Domínguez S. (2019). Editorial: beyond eating and body image disturbances: cultural, transcultural and accultural perspectives. Front. Psychol. 10:2601. doi: 10.3389/fpsyg.2019.02601, PMID: 31803125 PMC6873479

[ref12] Cervera S. Lahortiga F. Angel Martínez-González M. Gual P. Irala-Estévez J. D. Alonso Y. (2003). Neuroticism and low self-esteem as risk factors for incident eating disorders in a prospective cohort study. Int. J. Eat. Disord. 33, 271–280. doi: 10.1002/eat.10147, PMID: 12655623

[ref13] Chapman L. Cartwright-Hatton S. Thomson A. Lester K. J. (2021). Parental eating disorders: a systematic review of parenting attitudes, behaviours, and parent-child interactions. Clin. Psychol. Rev. 88:102031. doi: 10.1016/j.cpr.2021.102031, PMID: 34246839

[ref14] Culbert K. M. Sisk C. L. Klump K. L. (2021). A narrative review of sex differences in eating disorders: is there a biological basis? Clin. Ther. 43, 95–111. doi: 10.1016/j.clinthera.2020.12.003, PMID: 33375999 PMC7902379

[ref16] Estrella-Proaño A. Klein H. J. McCarthy S. M. (2022). Student wellness trends and interventions in medical education: a narrative review. Humanit. Soc. Sci. Commun. 9:92. doi: 10.1057/s41599-022-01105-8, PMID: 39310270

[ref17] Fekih-Romdhane F. Daher-Nashif S. Alhuwailah A. H. Al Gahtani H. M. Hubail S. A. Shuwiekh H. A. M.. (2022). The prevalence of feeding and eating disorders symptomology in medical students: an updated systematic review, meta-analysis, and meta-regression. Eat. Weight Disord. 27, 1991–2010. doi: 10.1007/s40519-021-01351-w, PMID: 35067859 PMC8784279

[ref9002] Garner D. M. (2004). Eating Disorder Inventory-3. Professional Manual. Psychological Assessment Resources, Inc., USA.

[ref18] Garner D. M. (2010). EDI-3: Inventarul tulburărilor de comportament alimentar-3: manual de specialitate: adaptarea și standardizarea EDI-3 pe populația din România. Cluj-Napoca: Editura ASCR.

[ref19] Ghamri R. A. Alahmari A. M. Alghamdi L. S. Alamoudi S. F. Barashid M. M. (2022). Prevalence and predictors of eating disorders: a cross-sectional survey of medical students at King Abdul-Aziz University, Jeddah. Pak. J. Med. Sci. 38, 1633–1638. doi: 10.12669/pjms.38.6.5033, PMID: 35991250 PMC9378390

[ref20] Guo J. Huang X. Zheng A. Chen W. Lei Z. Tang C.. (2022). The influence of self-esteem and psychological flexibility on medical college students' mental health: a cross-sectional study. Front. Psych. 13:836956. doi: 10.3389/fpsyt.2022.836956, PMID: 35651820 PMC9148951

[ref21] Halbeisen G. Braks K. Huber T. J. Paslakis G. (2022). Gender differences in treatment outcomes for eating disorders: a case-matched, retrospective pre–post comparison. Nutrients 14:2240. doi: 10.3390/nu14112240 35684040 PMC9183188

[ref22] Hoteit M. Mohsen H. Bookari K. Moussa G. Jurdi N. Yazbeck N. (2022). Prevalence, correlates, and gender disparities related to eating disordered behaviors among health science students and healthcare practitioners in Lebanon: findings of a national cross sectional study. Front. Nutr. 9:956310. doi: 10.3389/fnut.2022.956310 35928833 PMC9345498

[ref23] Hsu W. C. Fuh L. J. Liao S. C. (2024). Tickling the heart: integrating social emotional learning into medical education to cultivate empathetic, resilient, and holistically developed physicians. Front. Med. 11:1368858. doi: 10.3389/fmed.2024.1368858, PMID: 38500950 PMC10944992

[ref24] Hudson J. I. Hiripi E. Pope H. G. Jr. Kessler R. C. (2007). The prevalence and correlates of eating disorders in the National Comorbidity Survey Replication. Biol. Psychiatry 61, 348–358. doi: 10.1016/j.biopsych.2006.03.040, PMID: 16815322 PMC1892232

[ref25] Iorga M. Manole I. Pop L. Muraru I. D. Petrariu F. D. (2018). Eating disorders in relationship with dietary habits among pharmacy students in Romania. Pharmacy (Basel) 6:97. doi: 10.3390/pharmacy6030097, PMID: 30200444 PMC6164212

[ref26] Iorga M. Pop L. Muraru I. D. Ioan B. G. (2019). Screening the risk for eating disorders among medical dentistry students: a cross-sectional study. Rom. J. Oral Rehabil. 11, 102–110.

[ref27] Jahrami H. Dewald-Kaufmann J. Faris M. A. I. AlAnsari A. Taha M. AlAnsari N. (2020). Prevalence of sleep problems among medical students: a systematic review and meta-analysis. J. Public Health 28, 605–622. doi: 10.1007/s10389-019-01064-6

[ref28] Jahrami H. Saif Z. Faris M. A. Levine M. P. (2019). The relationship between risk of eating disorders, age, gender and body mass index in medical students: a meta-regression. Eat. Weight Disord. 24, 169–177. doi: 10.1007/s40519-018-0618-7, PMID: 30430465

[ref29] Jahrami H. Saif Z. Trabelsi K. Ghazzawi H. Pandi-Perumal S. R. Seeman M. V. (2024). An umbrella review and a Meta-analysis of Meta-analyses of disordered eating among medical students. Alpha Psychiatry 25, 165–174. doi: 10.5152/alphapsychiatry.2024.241515, PMID: 38798808 PMC11117415

[ref30] Jahrami H. Sater M. Abdulla A. Faris M. A. AlAnsari A. (2019). Eating disorders risk among medical students: a global systematic review and meta-analysis. Eat. Weight Disord. 24, 397–410. doi: 10.1007/s40519-018-0516-z, PMID: 29785631

[ref31] Joja O. (2001). History and current state of treatment for eating disorders in Romania. Eur. Eat. Disord. Rev. 9, 374–380. doi: 10.1002/erv.447

[ref32] Joja O. D. Nanu C. von Wietersheim J. (2015). Weight concerns and eating attitudes among Romanian students in comparison to German students and anorexia nervosa patients. Procedia. Soc. Behav. Sci. 187, 402–407. doi: 10.1016/j.sbspro.2015.03.075

[ref33] Kiss-Tóth E. Wasilewska M. Sopel O. Mandziuk M. Ladner J. Varga B.. (2018). Eating disorders in university students: an international multi-institutional study. Eur. J. Pub. Health 28:312. doi: 10.1093/eurpub/cky214.010 32549156

[ref34] Kovács T. (2007). Az evészavarok epicemiológiai vizsgálata romániai román, illetve magyar kultúrák tükrében [prevalence of eating disorders in the cultural context of the Romanian majority and the Hungarian minority in Romania]. Psychiatria Hungarica: A Magyar Pszichiatriai Tarsasag tudomanyos folyoirata 22, 390–396, PMID: 18421100

[ref35] Kovács Krizbai T. Szabó P. (2009). Az evészavarok epidemiológiai vizsgálata erdélyi román, magyar, illetve szász középiskolások körében [Prevalence of eating disorders in Romanian, Hungarian and Saxon secondary school students in Transylvania]. Psychiatria Hungarica: A Magyar Pszichiatriai Tarsasag tudomanyos folyoirata 24, 124–132. 19667423

[ref36] Ladner J. Lukács A. Brumboiu I. Ciobanu E. Croitoru C. Sasvári P.. (2019). Eating disorders among university students: a public health challenge. An European study. Eur. J. Pub. Health 29:563. doi: 10.1093/eurpub/ckz186.486

[ref37] Memon A. A. Adil S. E. Siddiqui E. U. Naeem S. S. Ali S. A. Mehmood K. (2012). Eating disorders in medical students of Karachi, Pakistan: a cross-sectional study. BMC. Res. Notes 5:84. doi: 10.1186/1756-0500-5-84, PMID: 22296613 PMC3395848

[ref38] Miclea Ș. Joja O. Albu M. (2011). “Studiul de adaptare şi standardizare a Inventarului tulburărilor de comportament alimentar (EDI-3) pe populaţia din România” in Manualul Inventarului tulburărilor de comportament alimentar EDI-3. ed. Garner D. M. (Cluj-Napoca: SC Cognitrom SRL).

[ref39] Mocanu V. (2013). Overweight, obesity, and dieting attitudes among college students in Romania. Endocrine Care 9, 241–248. doi: 10.4183/aeb.2013.241

[ref40] Nechita D. Nechita F. Motorga R. (2018). A review of the influence the anxiety exerts on human life. Rom J Morphol Embryol 59, 1045–1051. 30845283

[ref41] Nechita D. Nechita F. Strunoiu L. M. Albulescu D. M. Vasile D. L. (2019). Trait anxiety and coping in first year medical students. Rom J Morphol Embryol 60, 1059–1069. 31912124

[ref42] Petreanu M. Mirica A. Mirica R. Petreanu A. G. (2017). Eating behavior, mental health and degree of physical activity in medical students in Health and health psychology - icH&Hpsy 2017. European proceedings of social and behavioural sciences. Future Academy. eds. Bekirogullari Z. Minas M. Y. Thambusamy R. X., vol. 30, 228–235.

[ref43] Petunova S. Lazareva E. Zakharova A. Hartfelder D. Dulina G. Petunova Y.. (2022). Disordered eating behavior and body image in junior medical students. Eur. Psychiatry 63, S377–S378.

[ref44] Pike K. M. Hoek H. W. Dunne P. E. (2014). Cultural trends and eating disorders. Curr. Opin. Psychiatry 27, 436–442. doi: 10.1097/YCO.0000000000000100 25211499

[ref45] Pope H. G. Jr. Hudson J. I. Todd D. Y. Hudson M. S. (1984). Prevalence of anorexia nervosa and bulimia in three student populations. Int. J. Eat. Disord. 3, 45–51.

[ref46] Smith A. Bodell L. P. Holm-Denoma J. Joiner T. Gordon K. Perez M.. (2017). I don’t want to grow up, I’m a (gen X, Y, me) kid: increasing maturity fears across the decades. Int. J. Behav. Dev. 41, 655–662. doi: 10.1177/0165025416654302, PMID: 29225386 PMC5718623

[ref47] Soh N. L. Walter G. (2013). Publications on cross-cultural aspects of eating disorders. J. Eat. Disord. 1:4. doi: 10.1186/2050-2974-1-4, PMID: 24764527 PMC3776204

[ref48] Solly J. E. Chamberlain S. R. Lust K. Grant J. E. (2023). Binge-eating disorder in university students: high prevalence and strong link to impulsive and compulsive traits. CNS Spectr. 28, 61–69. doi: 10.1017/S1092852921000882, PMID: 34658319 PMC7614224

[ref49] Stackpole R. Greene D. Bills E. Egan S. J. (2023). The association between eating disorders and perfectionism in adults: a systematic review and meta-analysis. Eat. Behav. 50:101769. Advance online publication. doi: 10.1016/j.eatbeh.2023.101769, PMID: 37327637

[ref50] Stice E. Spangler D. Agras W. S. (2001). Exposure to media-portrayed thin-ideal images adversely affects vulnerable girls: a longitudinal experiment. J. Soc. Clin. Psychol. 20, 270–288. doi: 10.1521/jscp.20.3.270.22309

[ref51] Stice E. Whitenton K. (2002). Risk factors for body dissatisfaction in adolescent girls: a longitudinal investigation. Dev. Psychol. 38, 669–678. doi: 10.1037/0012-1649.38.5.669, PMID: 12220046

[ref52] Tetley A. Moghaddam N. G. Dawson D. L. Rennoldson M. (2014). Parental bonding and eating disorders: a systematic review. Eat. Behav. 15, 49–59. doi: 10.1016/j.eatbeh.2013.10.008, PMID: 24411750

[ref53] Túry F. Szabó P. Dukay-Szabó S. Szumska I. Simon D. Rathner G. (2020). Eating disorder characteristics among Hungarian medical students: changes between 1989 and 2011. J. Behav. Addict. 9, 1079–1087. doi: 10.1556/2006.2020.00078, PMID: 33245292 PMC8969710

[ref54] Túry F. Szabó P. Pászthy B. (2023). “Eating disorders in Eastern Europe” in Eating disorders. eds. Robinson P. Wade T. Herpertz-Dahlmann B. Fernandez-Aranda F. Treasure J. Wonderlich S. (Cham: Springer).

[ref55] Valladares-Garrido D. Quiroga-Castañeda P. P. Berrios-Villegas I. Zila-Velasque J. P. Anchay-Zuloeta C. Chumán-Sánchez M.. (2023). Depression, anxiety, and stress in medical students in Peru: a cross-sectional study. Front. Psych. 14:1268872. doi: 10.3389/fpsyt.2023.1268872, PMID: 38090694 PMC10715266

[ref56] Watson H. J. Davies H. L. Palmos A. B. (2023). “Genetics of eating disorders” in Eating disorders. eds. Robinson P. Wade T. Herpertz-Dahlmann B. Fernandez-Aranda F. Treasure J. Wonderlich S. (Cham: Springer).

[ref57] Zhang N. Ren X. Xu Z. Zhang K. (2024). Gender differences in the relationship between medical students’ emotional intelligence and stress coping: a cross-sectional study. BMC Med. Educ. 24:810. doi: 10.1186/s12909-024-05781-9, PMID: 39075473 PMC11285314

